# Perspectives and concerns of clients at primary health care facilities involved in evaluation of a national mental health training programme for primary care in Kenya

**DOI:** 10.1186/1752-4458-7-5

**Published:** 2013-01-23

**Authors:** Caleb Othieno, Rachel Jenkins, Stephen Okeyo, Julyan Aruwa, Jan Wallcraft, Ben Jenkins

**Affiliations:** 1 Department of Psychiatry, University of Nairobi, Nairobi, Kenya; 2 WHO Collaborating Centre (Mental Health), Institute of Psychiatry, King’s College London, London, UK; 3 Great Lakes University, Kisumu, Kenya; 4 University of Birmingham, Birmingham, UK; 5 Zacchaeus 2000 Trust, London, UK

## Abstract

**Background:**

A cluster randomised controlled trial (RCT) of a national Kenyan mental health primary care training programme demonstrated a significant impact on the health, disability and quality of life of clients, despite a severe shortage of medicines in the clinics (Jenkins et al. Submitted 2012). As focus group methodology has been found to be a useful method of obtaining a detailed understanding of client and health worker perspectives within health systems (Sharfritz and Roberts. Health Transit Rev 4:81–85, 1994), the experiences of the participating clients were explored through qualitative focus group discussions in order to better understand the potential reasons for the improved outcomes in the intervention group.

**Methods:**

Two ninety minute focus groups were conducted in Nyanza province, a poor agricultural region of Kenya, with 10 clients from the intervention group clinics where staff had received the training programme, and 10 clients from the control group where staff had not received the training during the earlier randomised controlled trial.

**Results:**

These focus group discussions suggest that the clients in the intervention group noticed and appreciated enhanced communication, diagnostic and counselling skills in their respective health workers, whereas clients in the control group were aware of the lack of these skills. Confidentiality emerged from the discussions as a significant client concern in relation to the volunteer cadre of community health workers, whose only training comes from their respective primary care health workers.

**Conclusion:**

Enhanced health worker skills conferred by the mental health training programme may be responsible for the significant improvement in outcomes for clients in the intervention clinics found in the randomised controlled trial, despite the general shortage of medicines and other health system weaknesses. These findings suggest that strengthening mental health training for primary care staff is worthwhile even where health systems are not strong and where the medicine supply cannot be guaranteed.

**Trial registration:**

ISRCTN 53515024.

## Background

Mental disorders are common in primary care across the world. A few specific interventions for single disorders or single client groups in low income countries have been evaluated but it remains challenging to scale up for all the usual mental disorders and client groups within existing human and financial resources [1,2]. At present, in African countries, on average only 0.7% of the health budget is spent on mental health services and national health budgets in Africa are often only around 10 USD per capita per year [3]. This means that there is a need to develop interventions that are affordable in the context of wider priorities and the current low resource situations prevailing in low income countries. There is evidence to suggest that general integration of specialist programmes into general health systems achieves better outcomes than more targeted disorder specific narrow integration [4].

Over the last 12 years, the Kenyan and Tanzanian governments have collaborated with the WHO Collaborating Centre, Institute of Psychiatry, and key stakeholders in national situation appraisal, including epidemiological surveys and stakeholder consultations and assisting development of national policies, strategic implementation plans and integration of mental health into national health sector reforms [5-7]. This previous work includes: a detailed situation appraisal of context, needs, resources, provision and outcomes using the mental health country profile [8]; a household survey exploring the conceptual model underlying the views of the general population about mental illness [9]; a focus group study of 60 traditional healers in Maseno District exploring their views of mental illness, aetiology and treatment [10]; a national survey of views of district level staff about mental illness [11]; studies in Tanzania and Kenya of attitudes of primary care staff about mental illness [12,13]; epidemiological surveys of mental disorders in two samples of 1000 people in urban Dar es Salaam, Tanzania [14-16], and in Maseno, a town in a rural district near Kisumu [17,18]; and adaptation of the World Health Organization primary care guidelines for Kenya and Tanzania.

Kenya, like a number of sub Saharan African countries, has a complex layered primary care system (described in detail elsewhere [6]), with the first level representing the community, the second level representing dispensaries, the third level health centres, and the fourth level district hospitals and their outpatient clinics. Since 2005, the Kenya Ministry of Health has conducted a programme to train 3000 level 2 and level 3 primary health care staff across Kenya in collaboration with the Kenya Medical Training College (KMTC), Kenya Psychiatric Association, and the WHO Collaborating Centre, Institute of Psychiatry, funded by Nuffield International Foundation and using a sustainable general health system approach. The content of training is informed by the above earlier qualitative and quantitative studies and closely aligned to the generic tasks of the health workers on child health, reproductive health, communicable and non-communicable diseases, and the training delivery integrated into the normal national training delivery system [19,20].

This mental health continuing professional development (CPD) training programme (known as the KMTC mental health CPD training programme) is a 5 day 40 hour programme conducted through theory, discussion, role plays and videos, and consists of 5 modules, with the first covering core concepts, the second core skills, the third common neurological disorders, the fourth psychiatric disorders (content based on the WHO primary care guidelines for mental health, Kenya version 2006) and the fifth module covering health sector and other sector system issues of policy; legislation; links between mental health and child health, reproductive health, HIV and malaria; roles and responsibilities; health management information systems; working with community health workers and with traditional healers; disaster planning (included after the 2007 Kenyan conflict, and consistent with the IASC guidelines [21]) and integration of mental health into annual operational plans. Each participant has to complete over 27 supervised role plays on different topics in the course of the week, and to observe and comment on 27 role plays conducted by colleagues.

The KMTC mental health CPD training course was developed and piloted in three courses in 2005; it was contributed to by colleagues from primary care, the Ministry of Health respective health programmes, University of Nairobi, and the professional and regulatory bodies for nurses and clinical officers. The course has been approved by the Kenya Nursing Council and the Clinical Officer Council of Kenya for 40 hours continuing professional development, now mandatory in Kenya. Role play scenarios are derived from real Kenyan clinical cases.

In 2010 the research project conducted a phase 2 exploratory trial as a pilot cluster RCT testing the effect of an affordable low cost training intervention - integrated with the national health sector reforms - intended to be accompanied by routine supervision from district staff, routine availability of medicines in the clinics, and provision during the training programme of WHO primary care guidelines adapted for Kenya: (i) on the competencies of primary care staff to recognise mental disorders, treat and make appropriate referrals to the scarce specialist service; (ii) on recovery (improved health and social outcomes and quality of life) of clients. The trial was conducted in a real clinical field setting, using local trainers (who had been trained in 2006, received a refresher course in 2009 and had delivered several such courses per year since 2005) to train health staff in the intervention group. The project did not exert any special influence on the usual local availability of medicines, district supervision or local health management information systems. During the course of the trial Kenya, and especially Nyanza Province, experienced a severe shortage of medicines, and this was reflected in the research findings on medicine availability in the clinics participating in the trial. Nonetheless, the trial found significant improvement in the clients of the trained health workers in the intervention group compared with those in the control group [22]. Therefore, at the request of the funder (the UK Department for International Development) we conducted focus groups with some health workers and clients from the trial to better understand their perspectives and experiences. Focus group methodology has been found to be an effective way to explore health worker and client views within health system contexts [23]. This paper reports the experiences of the clients, and an accompanying paper reports the experiences of the health workers.

## Methods

During the randomised controlled trial, we had followed up for 3 months 12 clients positive on the General Health Questionnaire in each of 100 clinics. For the purpose of the focus group study we contacted one such client from each of 10 intervention clinics, and 10 control clinics, selected at random. We invited them to take part in one of four ninety minute focus groups (10 clients from the intervention clinics and 10 clients from the control clinics). We paid respondents’ transport and subsistence for the day they came for the interviews. No additional payments were made. There was no communication about the study between the different groups of participants before their respective focus groups were held.

### Ethical considerations, explanation and consent

Ethical approval was obtained from Kings College London ethical committee and the University of Nairobi ethics committee. An explanatory information sheet was given to each client in English and Kiswahili, and read out to those who could not read. We explained verbally in English, Kiswahili and Luo to the participants the purpose of the meeting and asked for their individual consents. They were reassured of confidentiality and told that we wanted to learn from their experiences. We then assigned each participant an identification number. They were told that the conversations would be taped but they would not be identified by name.

The focus group discussions were held in August 2011, eight months after the end of the randomised controlled trial, during a residential two days in a small hotel at Chulaimbo, near Kisumu, which was easily accessible by all those invited. They were conducted by CO assisted by RJ, and each lasted 90-120 minutes. Discussions were conducted mainly in English, but also in the local language where participants could not understand English or found it easier to express themselves in Luo. A voice recording of all the sessions was made, and both RJ and JA took written notes which were used to help clarify the transcript material where necessary.

### Instruments

The discussions were guided by the following questions:

1. Could you describe to us your experience in attending the health centre?

2. Describe the response you get when you present an emotional problem to the health worker.

3. What is your view regarding the communication between you and the health workers:

4. Do you feel understood?

5. Do you find the health worker helpful?

6. How much time do you spend with the health worker?

7. Are you given an opportunity to ask questions?

8. Do you get a satisfactory explanation about your condition?

9. What further information would you like?

10. What treatment options did the health worker provide?

11. Were you referred anywhere else?

12. Please rate your satisfaction with the services

13. Have you noticed any changes in the way you are treated at the health centre?

14. What impact has it had on your health?

15. Do you have any other concerns?

The guideline questions were used to structure the conversation. However the clients were allowed to discuss issues which they felt were important to them. Thus issues that were not explicitly spelt out in the guideline questions came up. In the analysis as stated common themes were grouped together. For example questions 2 – 9 were brought up issues concerning communication. Questions on satisfaction with the health services, other concerns (questions 12 – 15) led to discussions on confidentiality, and the need for more frequent home visits. Treatment options and referral issues (questions 10 – 11) are discussed under coordination. The patient’s experiences of taking emotional problems to the clinics (1) brought up issues of recognition of mental health problems and treatments offered.

### Data analysis

The recordings of the discussions were transcribed and translated into English where necessary. The voice recordings and the transcripts were analysed for common themes emerging in response to the guideline questions. Matrices were created to help facilitate the comparison of text across the different categories of informant.

## Results of client focus group discussions

All those invited attended, namely 10 clients from the intervention group and 10 from the control group. Each was from a different health centre.

### Health education

The patients in both intervention and control groups reported receiving general educational talks in the clinics, which involved mainly nutrition, HIV information and hygiene:

*“What I can say about our clinic is that we are being taught. On the clinic days all patients are assembled together and taught about food …we are taught we should not just eat fish and meat but also consider having vegetables.”* Client 3, control group.

They requested to be given more education on mental health:

*“I just wanted to ask, this stress which often comes to me, why does it come, because I think a lot?”* Client 6, control group.

### Communication skills

Clients in the intervention group reported a welcoming reception in the clinic while the control group described poor reception and even rudeness. The patients in the control group were generally less satisfied with the treatment offered in the health facilities. In particular they were dissatisfied with the way they were handled by the health workers.

*“We would like to be talked to softly and educated.”* Client 3, control group.

*“Patients should not be neglected or avoided even when they are terminally ill.”* Client 5, control group.

The clients from the intervention clinics had better relations with the health workers and could easily talk to them:

*“In my area it is easy for me to discuss with the health workers. They welcome us and make us at home, so it is easy to explain my problem.”* Client 9, intervention group.

Some clients in the control group reported major concerns with the way they were communicated with during child birth:

*“Firstly, when you see a woman who is in labour struggling, you do not tell her shocking things. For example, if the baby is stuck next to her heart and these are things which frighten us. Hence when you go to the clinic, you feel you will be dead. You (nurse) should just keep quiet even when the child is coming out by the legs. You (nurse) should just tell her ‘keep cool mama things will be alright’.”* Client 4, control group.

*“Another one can cheat women that ‘mama the time is not yet just be patient there’ and yet the waters have started pouring out*.*”* Client 6, control group.

*“Sometimes you are harassed, beaten and even abused and these things make you feel bad.”* Client 8, control group.

*“When I was taken to Bondo in 2006 for a blood transfusion, the veins could not be traced and the doctor told me, ‘woman, you have no luck’. If you are told you are not lucky, you are automatically going to die. You will die because you have lost hope of living.”* Client 4, control group.

There were also complaints from clients in the control group about the way people with HIV were treated:

“*That sister when she diagnosed you that you have the disease (HIV), and when you go to the hospital, she can even abuse you that you have AIDS. When she finds out that you are not taking the ARVs accordingly, she also abuses you in front of others, even if you did not want other people to hear … There are others also who harass people.”* Client 7, control group.

### Recognition of adult mental health problems

These were poorly recognised in the control group and one patient narrated how she had taken anti-malarial tablets and paracetamol repeatedly for headache without improvement. When she consulted a doctor, she was tested and treated for typhoid and she is still unwell. Others described common mental disorders which had not been offered appropriate treatment or referral. One client wanted to know why she had stress, she said *“Why does it come and make me think a lot?… When many things come my head stops functioning”.* Another client experiences frequent headaches, right in the middle of the head. It is aggravated by noise. She also feels pains all over her body. She said, *“I behave like someone already dead”.*

### Recognition of children’s mental health problems

Problems identified by the intervention group included school refusal due to school phobia or anxiety; somatisation; substance abuse – illicit brew (chang’aa) and cannabis (bhang). The clients in both groups requested practical information on how they could cope with their children:

*“Some children could be fearing school. Some would pretend that they are sick. The orphans might be mistreated by their guardian which may affect them. Such children may pretend to be sick. Such a child may need to see the doctor.”* Client 8, intervention group.

### Clients views on areas where clinic improvement is needed

More areas needing improvement were highlighted by clients in the control group than in the intervention group.

#### (a) Communication skills

The patients in the control group wanted better doctor patient relationships, respect for patients, not to be blamed, abused and assaulted by health care staff, or even chased away by them. The health care staff should learn how to break bad news to patients, involve them more in management especially when making referrals, and not neglect patients when they are seriously ill. Patients should always be given hope.

*“They should be taught on how to speak gently to the patients by telling them to put God first in their treatment regardless of the patients’ sicknesses. They should not tell the patients things which give them shocks such as deaths which can frighten them.”* Client, control group.

*“If I may add a bit on the doctors following what my other sister has said, ‘beatings’. There are some people who do not know how to talk to patients. Patients should not be given sad news but they should be talked to politely by telling them to believe in their God because he is a better healer than the doctors. Whether you are going to die you should be talked to in a polite manner rather just neglecting you.”* Client, control group.

In addition they would like more help in situations where they cannot afford to buy medication.

*“At times, patients do not have adequate finances to buy medicine and when they go on that other side (the government clinic pharmacy) they are again chased away. So according to me the doctors should try to find out in a softer approach how much money the patients have. Supposing they do not have sufficient money, can they be allowed or helped?”* Client 3, control group.

#### (b) Coordination

Clients in the control group felt that more coordination was needed when making referrals so that they are not sent to and from the pharmacies, only to find that no medicines were available.

*“There are times when you go to a doctor, he prescribes medicines and he gives you prescription to take to the pharmacist. You go to the pharmacist and you are told there are no medicines. What I am wondering, does the prescribing doctor know that it is there or not?”* Client, control group.

*“Doctors should consider the socio-economic status of the patients when prescribing medications and offer appropriate help to needy patients.”* Client, control group.

#### (c) Home visits

The patients in the intervention group suggested that they should have more frequent home visits by the cadre of volunteer community health workers as it was easier to discuss some issues when they were at home.

“*Community health workers (CHWs) should visit more frequently to know our problems. By doing this, we could discuss more. It is easier when they come to our home*.” Client, intervention group.

#### (d) Confidentiality

However, they felt that the community health workers should observe confidentiality.

“*There are some CHWs who are not experienced enough to use confidentiality. This may lead to stigma because these people may start spreading*.”

In cases where confidentiality was not reliably observed, patients preferred travelling to hospitals far from home where they could not be easily identified by their community members.

“Like in my case, I discussed my problems with the community health workers and it spread out. Now I feel more free explaining to the hospitals than the community health workers.”

## Discussion

This is the first qualitative focus group study of clients involved in a randomised controlled trial of primary care training on mental health in Kenya. The study was conducted in Nyanza province, Kenya, in the public sector primary care system, and the study findings are limited to this group, although are likely to have wider relevance for Kenyan primary care as a whole. Further limitations of the study include the fact that most of the research team for the focus group study were also involved in the randomised controlled trial. The use of both clients and health workers (see accompanying paper [24]) affords some triangulation of the findings.

From the focus group discussions it seems that the clients in clinics where staff had been trained in the KMTC mental health programme experienced better reception, communication and overall support than clients in clinics where staff had not been trained.

Since the drug supply was poor in both the control and the intervention groups it is likely that these enhanced psychosocial skills in the health workers, noticed by both themselves (see accompanying paper [24]) and their clients, are the factors that contributed to better recovery in the clients in the intervention group, compared to those in the control clinics in the randomised controlled trial reported elsewhere [22].

These study findings concur with those found in a randomised controlled trial of the same training programme in Iraq, where research observers found better diagnosis and communication skills in staff trained in the program, compared to controls [25].

These findings support the value of strengthening mental health training for primary care staff; the value of including major emphasis in the training on the core generic psychosocial skills, and on the links between mental health, malaria, HIV, child and reproductive health; and indicate that such training is worthwhile even where health systems are not strong and where the medicine supply cannot be guaranteed. The findings suggest that such training would benefit from more emphasis on client confidentiality especially in relation to the volunteer cadre of community health workers, whose training is generally delivered by primary care health workers in regular weekly talks.

When comparing this Kenyan experiential data with that reported by clients and primary care doctors and nurses in the UK, where there is also an agenda to take people and their lived experience into account to deliver more effective services [26,27], UK focus groups have found that clients see primary care as the corner stone of their health care [28,29] but, like the Kenyan clients, perceive that primary care professionals give more priority to physical illness than to mental illness. UK clients also feel that their primary care staff are more concerned with identifying risk and stabilising illness rather than helping recovery. UK clients do not have to contend with shortage of medicines, nor do they generally report verbal and physical abuse. Nonetheless there is still a mismatch of expectations and concerns at the primary care level between clients and health workers in the UK. Recent NICE (National Institute for Health and Clinical Guidance) guidelines and quality standards on patient experience has set out the principle that high quality client experience should be at the heart of good clinical care [30], and the data reported here indicates that clients consider this principle to be just as relevant in Kenya as it is elsewhere.

## Conclusion

These focus group discussions suggest that the clients in the intervention group noticed and appreciated enhanced communication, diagnostic and counselling skills in their respective health workers, while clients in the control group were aware of the lack of these skills. These enhanced generic health worker skills conferred by the mental health training programme may be at least partly responsible for the significant improvement in outcome of patients in the intervention clinics where staff had received the training programme, compared to the control group, despite the general shortage of medicines and other health system weaknesses.

## Competing interests

The authors declare that they have no competing interests.

## Authors’ contributions

CO thieno chaired the focus group discussions, supplied the transcription of data and the main analysis of findings, and wrote the first draft of the paper. RJ was responsible for overall design of the study, obtained funding, co-led the focus groups with Caleb Othieno, and wrote the subsequent drafts of the paper. SO supervised the local implementation of the study design. JA coordinated the invitations of client and health workers, and took notes of the focus group discussions. JW checked the qualitative methodology and contributed to UK comparisons of the discussion. BJ advised on the specific content of the focus groups, and organised the audio taping of the discussions. All authors contributed to and approved the final version of the paper.

## Funding

UK Department of International Development.
